# Hepatic encephalopathy increases the risk of hip fracture: a nationwide cohort study

**DOI:** 10.1186/s12891-020-03811-3

**Published:** 2020-11-26

**Authors:** Kuang-Ting Yeh, Tzai-Chiu Yu, Ru-Ping Lee, Jen-Hung Wang, Kuan-Lin Liu, Cheng-Huan Peng, Hao-Wen Chen, Ing-Ho Chen, Chung-Yi Hsu, Wen-Tien Wu

**Affiliations:** 1 Department of Orthopedics, Hualien Tzu Chi Hospital, Buddhist Tzu Chi Medical Foundation, Hualien, Taiwan; 2 School of Medicine, Tzu Chi University, Hualien, Taiwan; 3 Institute of Medical Sciences, Tzu Chi University, Hualien, Taiwan; 4 Department of Medical Research, Hualien Tzu Chi Hospital, Buddhist Tzu Chi Medical Foundation, Hualien, Taiwan; 5 Graduate Institute of Clinical Medical Science, China Medical University, Taichung, Taiwan

**Keywords:** Nationwide cohort study, Hepatic encephalopathy, Osteoporotic hip fracture, Propensity score, Kaplan–Meier method

## Abstract

**Background:**

Osteoporotic hip fracture is a common general health problem with a significant impact on human life because it debilitates the patients and largely decreases their quality of life. Early prevention of fractures has become essential in recent decades. This can be achieved by evaluating the related risk factors, as a reference for further intervention. This is especially useful for the vulnerable patient group with comorbidities. Hepatic encephalopathy (HE), a major complication of liver cirrhosis, may increase the rate of falls and weaken the bone. This study evaluated the correlation between hepatic encephalopathy and osteoporotic hip fracture in the aged population using a national database.

**Methods:**

This retrospective cohort study used data from Taiwan’s National Health Insurance Research Database between 2000 and 2012. We included people who were older than 50 years with hepatic encephalopathy or other common chronic illnesses. Patients with and without hepatic encephalopathy were matched at a ratio of 1:4 for age, sex, and index year. The incidence and hazard ratios of osteoporotic hip fracture between the both cohorts were calculated using Cox proportional hazard regression models.

**Results:**

The mean age of the enrolled patients was 66.5 years. The incidence ratio of osteoporotic hip fracture in the HE group was significantly higher than that in the non-HE group (68/2496 [2.7%] vs 98/9984 [0.98%]). Patients with HE were 2.15-times more likely to develop osteoporotic hip fractures than patients without HE in the whole group. The risk ratio was also significantly higher in female and older individuals. The results were also similar in the comorbidity subgroups of hypertension, diabetes mellitus, hyperlipidemia, senile cataract, gastric ulcer, and depression. Alcohol-related illnesses seemed to not confound the results of this study.

**Conclusions:**

HE is significantly associated with an increased risk of osteoporotic hip fractures, and the significance is not affected by the comorbidities in people aged more than 50 years. The cumulative risk of fracture increases with age.

## Background

Osteoporosis, a complication of liver cirrhosis, is strongly correlated with the incidence of osteoporotic fractures [1]. Osteoporotic hip fractures (OHFx) often occur due to low-energy mechanisms, such as falling on the ground with sudden changes in position, in elderly people or women who undergo surgical menopause [2]. OHFx is a common general health problem with a significant impact on human life because it debilitates the patients and largely decreases their quality of life. Early prevention of the fracture, which has become essential in recent decades, can be achieved by evaluating the related risk factors, as a reference for further intervention. This is especially useful for the vulnerable patient group with comorbidities. In patients with chronic liver disease, hepatic encephalopathy (HE) is one of the most common conditions that cause disability and irreversible brain injury [3, 4]. The rate of mortality is high in patients with liver cirrhosis and complications of severe HE, even in the first year [5]. Due to our improved understanding of the HE progression and improvements in the resuscitation and evolved support systems, the life expectancy of patients with HE has grown [6]. The occurrence of OHFx in patients with HE results in severe disability and a high mortality rate; HE further complicates the treatment of OHFx, potentially placing a considerable medical burden on society [7, 8].

Due to the increasing life span of patients with liver cirrhosis and HE, it becomes important to assess the association between osteoporosis-related fractures and HE. However, few large-scale study has investigated the relationship between HE and OHFx by comparing patients with and without HE. The present retrospective cohort study aimed to investigate the correlation between the risk of OHFx and HE in people aged more than 50 years by using data from Taiwan’s National Health Insurance (NHI) program.

## Methods

### Data sources

The NHI Research Database (NHIRD) contains health-related data of nearly the entire population of Taiwan. The NHI program, which was launched in 1995, provides all-round medical care including outpatient and inpatient care to approximately 99% of the 23.74 million citizens of Taiwan. The Longitudinal Health Insurance Database 2000 (LHID2000), a subset of NHIRD, contains the data of 1 million beneficiaries randomly selected from the Registry for beneficiaries of the year 2000. These random samples found in LHID2000 have been confirmed by the NHIRD to be representative of Taiwanese residents. For each beneficiary, a unique identification number is used to link all insurance information and health care records. In the NHIRD, diseases are defined according to the International Classification of Diseases, Ninth Revision, Clinical Modification (ICD-9-CM) codes. This study was approved by the Research Ethics Committee of China Medical University and Hospital in Taiwan (CMUH-104-REC2–115).

### Sample design

**Fig. 1:**
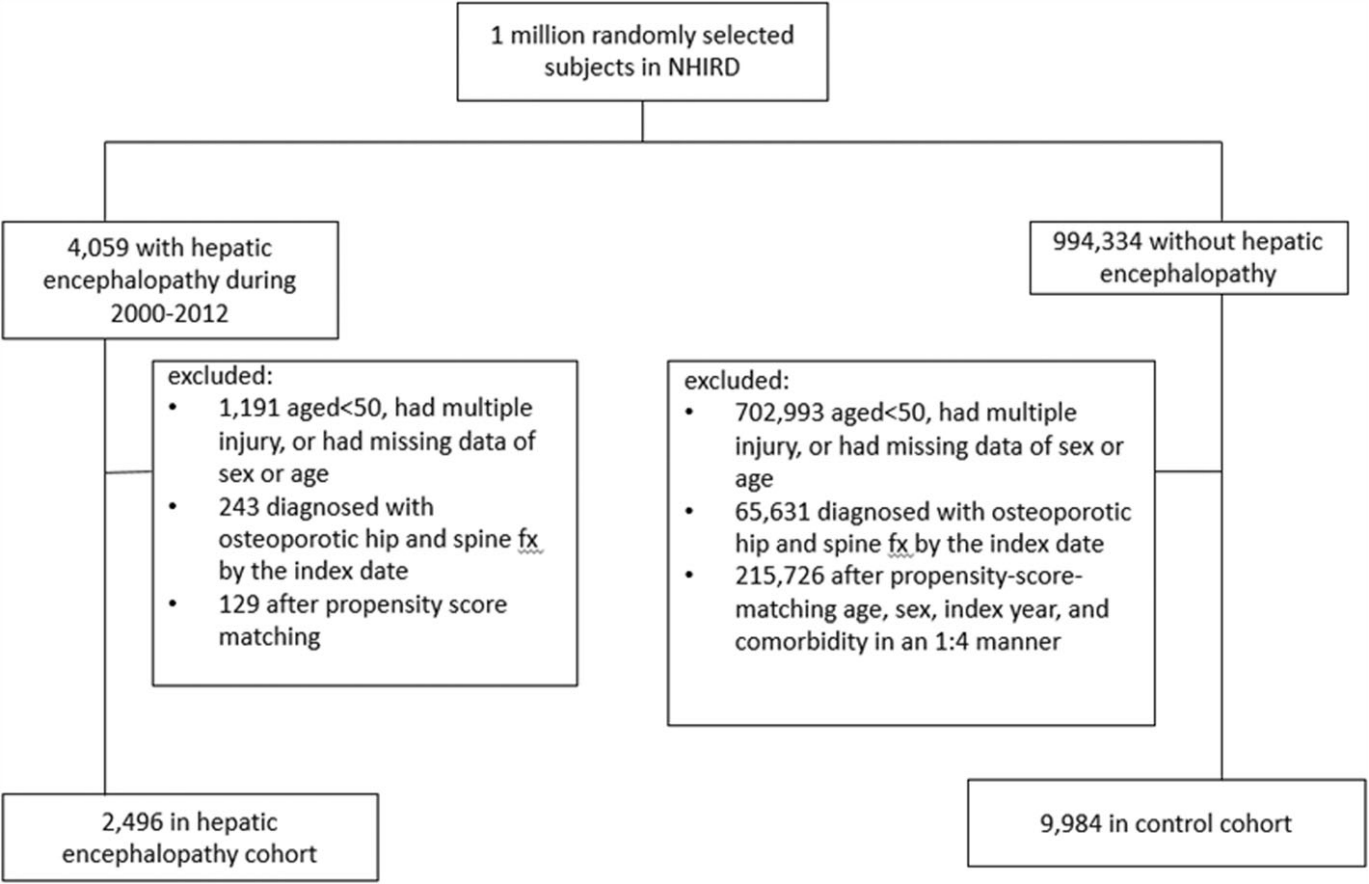
Flow chart of the process for establishing the hepatic encephalopathy cohort and control group on the basis of the National Health Insurance Research Database

From the NHIRD, we identified and enrolled people aged more than 50 years between 2000 and 2012 in this cohort study. Patients with HE (ICD-9-CM code 572.2) and those without HE were included in the case and control cohorts, respectively. The study outcome was the incidence of OHFx (ICD-9-CM codes 733.00–733.09, and 820.0–820.9). The index date was the first date of HE diagnosis for the case cohort and a random date as the index year for the control cohort. We excluded patients who developed with OHFx before the index date, had multiple injuries (ICD-9-CM code 959.99), or had missing information on sex or age. The follow-up period on patients lasted until the diagnosis of OHFx, death, withdrawal from the insurance, or the end of 2013. According to the logistic regression model, we matched age, sex, index year, and comorbidities between the case and control cohorts by using propensity scores at a ratio of 1:4. We examined the distribution of sex, age, and comorbidities, namely hypertension (ICD-9-CM codes 401–405), diabetes mellitus (DM; ICD-9-CM code 250), alcohol-related illnesses (ICD-9-CM 291, 303, 305.0, 571.0, 571.1, 571.2, 571.3, 790.3, A215, and V11.3), hyperlipidemia (ICD-9-CM code 272), senile cataract (ICD-9-CM codes 366.10–366.19), gastric ulcer (ICD-9-CM codes 531.0–531.9), cholangitis (ICD-9-CM code 576.1), and depression (ICD-9-CM codes 296.2, 296.3, 296.82, 300.4, 309.0, 309.1, and 311) between the case and control cohorts. The entire design and screening process are presented as a flowchart in Fig. 1.

### Statistical analysis

The standardized mean difference (SMD) on strata of sex, age, comorbidity, and follow-up period were applied for further analysis. The incidence rate (IR) was defined as the number of events divided by person-years. Crude hazard ratios, adjusted hazard ratios (aHRs), and 95% confidence intervals (95% CIs) were calculated based on the multivariable Cox proportional hazard regression model adjusted for sex, age, and comorbidities. The Kaplan–Meier method was used to determine the cumulative incidence of OHFx in patients with and without HE, and the log-rank test was used to examine its significance. Statistical analysis was performed using SAS 9.4 software (SAS Institute, Cary, NC, USA); *P* < 0.05 was considered to indicate statistical significance.

## Results

A total of 2496 patients with HE and 9984 patients without HE were included. The very small values of SMD show that the demographic data had great similarities between the two groups, including the distributions of sex, age, and associated comorbidities (Table 1).

**Table 1:** Baseline characteristics in patients with and without hepatic encephalopathy

	Hepatic encephalopathy		Standardized mean difference
	No (*n* = 9984)	Yes (*n* = 2496)	
	n (%)	n (%)	
Sex			
Female	3708 (37.1)	943 (37.8)	0.01
Male	6276 (62.9)	1553 (62.2)	0.01
Age, years			
50–64	4427 (44.3)	1163 (46.6)	0.05
65–79	4425 (44.3)	1081 (43.3)	0.02
80+	1132 (11.3)	252 (10.1)	0.04
Mean (SD)	66.5 (10.4)	66.7 (10.1)	0.02
Comorbidity			
Hypertension	6125 (61.4)	1473 (59.0)	0.05
Diabetes mellitus	4446 (44.5)	1073 (43.0)	0.03
Hyperlipidemia	2847 (28.5)	685 (27.4)	0.02
Senile cataract	2832 (28.4)	686 (27.5)	0.02
Gastric ulcer	4285 (42.9)	1053 (42.2)	0.02
Cholangitis	342 (3.43)	114 (4.57)	0.06
Chronic renal failure	0	0	
Depression	1019 (10.2)	261 (10.5)	0.01
Alcohol-related illnesses	2131 (21.3)	537 (21.5)	0.004

A standardized mean difference ≤ 0.10 indicates a negligible difference between the two cohorts

The incidence ratio of hip fracture in the HE group was 68/2496 (2.7%), which was significantly higher than that in the non-HE group, which was 98/9984 (0.98%). Patients with HE were 2.15-times more likely to develop OHFx than those without HE (IR per 1000 person-years = 20.4 vs 9.8, *P* < 0.001) (Table 2). Female patients with HE were 2.25-times more likely to develop OHFx than female patients without HE, while male patients with HE were 2.00-times more likely to develop OHFx than male patients without HE (*P* < 0.001). HE patients in the age range of 50–64 and 65–79 years were respectively 3.57- and 2.51-times more likely to develop OHFx than their non-HE counterparts (*P* < 0.001).

**Table 2:** Incidence and hazard ratios of osteoporotic hip fracture between patients with and without hepatic encephalopathy

	Hepatic encephalopathy										
	No			Yes							
	Event	PY	Rate^a^	Event	PY	Rate^a^	cHR	(95% CI)	aHR	(95% CI)	*P*-value
Overall	575	58,393	9.8	68	3337	20.4	2.15	((1.67,2.78)^***^	2.36	(1.82,3.05)^***^	< 0.001
Sex											
Female	332	22,154	15.0	41	1278	32.1	2.25	((1.62,3.13)^***^	2.39	(1.71,3.34)^***^	< 0.001
Male	243	36,239	6.7	27	2058	13.1	2.00	((1.34,3.00)^***^	2.24	(1.49,3.36)^***^	< 0.001
Age, years											
50–64	105	28,340	3.7	24	1972	12.2	3.57	((2.27,5.60)^***^	2.87	(1.81,4.54)^***^	< 0.001
65–79	350	25,837	13.5	34	1144	29.7	2.51	((1.76,3.60)^***^	2.42	(1.69,3.47)^***^	< 0.001
80+	120	4216	28.5	10	221	45.3	1.52	((0.79,2.92)	1.49	(0.78,2.88)	0.056
Comorbidity											
Hypertension	408	32,778	12.4	49	1910	25.7	2.18	((1.62,2.95)^***^	2.44	(1.80,3.31)^***^	< 0.001
Diabetes mellitus	277	23,738	11.7	30	1348	22.3	1.96	((1.34,2.87)^***^	2.09	(1.42,3.07)^***^	< 0.001
Hyperlipidemia	145	14,205	10.2	25	1081	23.1	2.38	((1.55,3.65)^***^	2.68	(1.74,4.15)^***^	< 0.001
Senile Cataract	204	13,964	14.6	21	828	25.3	1.77	((1.13,2.80)^*^	2.02	(1.28,3.20)^**^	< 0.001
Gastric Ulcer	289	23,761	12.2	36	1432	25.1	2.07	((1.46,2.95)^***^	2.31	(1.62,3.30)^***^	< 0.001
Cholangitis	27	1595	16.9	2	109	18.4	1.18	((0.27,5.07)	1.10	(0.23,5.32)	0.078
Depression	66	5053	13.1	11	443	24.8	2.02	((1.06,3.84)^*^	2.19	(1.12,4.20)^*^	0.048
Alcohol-related illnesses	79	10,429	7.6	13	1117	11.6	1.57	((0.87,2.83)	1.91	(1.03,3.49)^*^	0.047

*aHR* adjusted hazard ratio, *cHR* crude hazard ratio, *PY* person-years

*P < 0.05, **P < 0.01, ***P < 0.001

rate^a^, incidence rate per 1000 person-years

HE patients with cholangitis bear the same risk of hip fracture as non-HE patients with cholangitis (*P* = 0.078). HE patients with the other comorbidities, including HTN, hyperlipidemia, gastric ulcer, depression, DM, senile cataract, and alcohol-related illnesses were respectively 2.44-, 2.68-, 2.31-, 2.19-, 2.09-, 2.02-,1.91-times more likely to develop OHFx than their non-HE counterparts (*P* < 0.05) (Table 2).

**Fig. 2:**
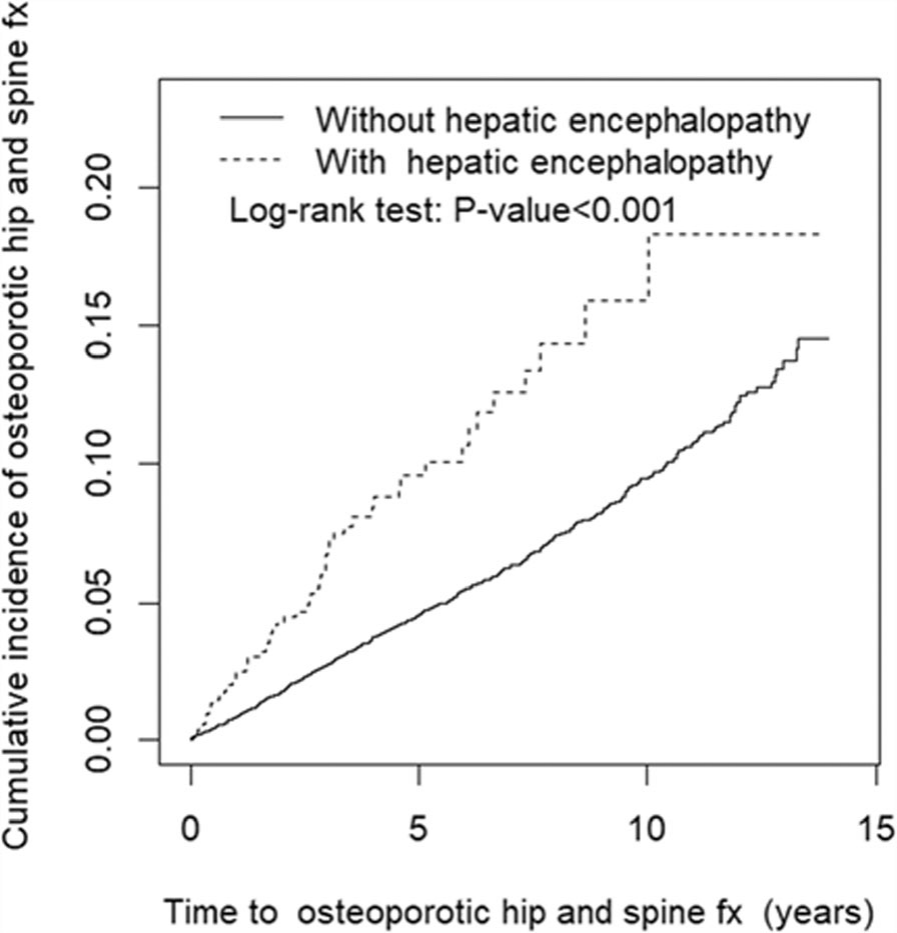
Cumulative incidence of osteoporotic hip fractures of patients with and without hepatic encephalopathy

The cumulative incidence of OHFx differed significantly between patients with and without HE (log-rank test: *P* < 0.001) (Fig. 2).

## Discussion

This study revealed that the incidence of OHFx was significantly higher in elderly HE patients (aged more than 50 years) than in individuals without HE. The cumulative incidence of OHFx was also significantly higher in patients with HE, even though the follow-up period was shorter in the HE group than in the non-HE group, which may be due to a shorter life span related to HE as a comorbidity.

Many studies have found that patients with hepatic failure have an increased risk of osteoporosis; the pathogenesis of bone loss and osteoporosis in patients with hepatic failure is complex and multifactorial [7, 9]. A study reported that low levels of insulin-like growth factor 1 in patients with advanced liver cirrhosis may aggravate bone remodeling and maintenance of bone mass in elderly patients, causing fragility fractures [10, 11]. In addition, patients with HE have demonstrated poor cognitive function and often develop psychiatric illnesses, which may increase the risk of sustaining an injury due to a fall [5].

In this study, patients with HE were 2.15-times more likely to develop OHFx than those without HE. This trend was similar in the subgroup of HTN, hyperlipidemia, gastric ulcer, depression, DM, senile cataract, and alcohol-related illnesses, except for the subgroup of cholangitis. It indicated that HE is a crucial risk factor for OHFx. In addition, the risk increased with time, based on the log-rank test. Patients without the comorbidities listed in Table 2 had a similar risk of OHFx, but patients with cholangitis, senile cataract, alcohol-related illnesses, and DM in the case group had a lower risk of OHFx (less than 2 times) than those in the control group. Thus, these four comorbidities may significantly increase the risk of OHFx. Hyperbilirubinemia can be found in cholangitis and has been shown to impair osteoblast proliferation and result in decreased bone formation [12–14]. A recent study found that senile cataract is independently associated with an increased risk of osteoporosis and fractures and that the reasons for this association are multifactorial, mainly relating to aging and a high possibility of sustaining a fall injury [15]. A study reported that women aged between 67 and 90 years who consumed an average of more than 3 oz of alcohol per day (equivalent to six drinks) had higher bone loss than women who had less alcohol intake [16]. In an animal study, older rats who were administered alcohol were found to have deficiencies in bone volume and density. Both type 1 and type 2 DM are associated with decreased bone strength, and alterations in bone quality play a major role in the pathogenesis of fragility fractures [17]. The insulin growth factor pathway, one of the complex pathways involved in the relationship between DM and bone fragility, mediates the accumulation of advanced glycation end products in bone collagen. This results in microangiopathy and increased fat content in the bone marrow [18]. The mechanism underlying the interaction between HE and DM and senile cataract warrants further investigation.

The advantage of our study is the large sample size. Selection and nonresponse biases may have been minimized due to the comprehensive coverage of the NHI system (> 95% of the Taiwanese population). This study also has some limitations. First, we could not determine the severity of hepatic damage because the NHIRD does not provide information regarding symptoms. Second, important sources of bias such as lifestyle factors, personal characteristics, and biochemical data, could not be obtained. Third, the findings of this study may not be directly generalizable to Caucasian or African populations as our results are based on data from Taiwan’s NHI. Even though, this study still provided the correlation between HE, OHFx and the comorbidities. We believe that aggressive treatment of osteoporosis and complete geriatric care strategies for older patients with HE should be routinely considered based on our study results. The interaction between the related risk factors of OHFx for the patients with HE should be studied in the future.

## Conclusions

HE appears to be a crucial risk factor for OHFx in people aged more than 50 years based on the findings of this nationwide cohort study, and this significance can be found similarly in the subgroups of HTN, DM, hyperlipidemia, senile cataract, gastric ulcer, and depression, which all play an important role in the pathogenesis of OHFx. This is an association retrospective cohort study, and prospective research is needed to derive more promising conclusions.

## Data Availability

Data are available from the NHIRD published by Taiwan NHI Bureau. Due to legal restrictions imposed by the government of Taiwan in relation to the “Personal Information Protection Act”, data cannot be made publicly available. Requests for data can be sent as a formal proposal to the NHIRD (http://nhird.nhri.org.tw).

## Abbreviations

95% CI: 95% confidence interval
aHR: adjusted hazard ratio
DM: diabetes mellitus
HE: hepatic encephalopathy
HTN: hypertension
ICD-9-CM: International Classification of Diseases, Ninth Revision, Clinical Modification
IR: incidence rate
LHID2000: Longitudinal Health Insurance Database 2000
NHI: National Health Insurance
NHIRD: NHI Research Database
OHFx: Osteoporotic hip fractures
SMD: standardized mean difference
